# An exome sequencing strategy to diagnose lethal autosomal recessive disorders

**DOI:** 10.1038/ejhg.2014.120

**Published:** 2014-06-25

**Authors:** Sian Ellard, Emma Kivuva, Peter Turnpenny, Karen Stals, Matthew Johnson, Weijia Xie, Richard Caswell, Hana Lango Allen

**Affiliations:** 1 Institute of Biomedical and Clinical Science, University of Exeter Medical School, Exeter, UK; 2 Department of Molecular Genetics, Royal Devon and Exeter NHS Foundation Trust, Exeter, UK; 3 Department of Clinical Genetics, Royal Devon and Exeter NHS Foundation Trust, Exeter, UK

## Abstract

Rare disorders resulting in prenatal or neonatal death are genetically heterogeneous. For some conditions, affected fetuses can be diagnosed by ultrasound scan, but this is not usually possible until mid-gestation. There is often limited fetal DNA available for investigation. We investigated a strategy for diagnosing autosomal recessive lethal disorders in non-consanguineous pedigrees with multiple affected fetuses. Exome sequencing was performed to identify genes where each parent is heterozygous for a rare non-synonymous-coding or splicing variant. Putative pathogenic variants were tested for cosegregation in affected fetuses and unaffected siblings. In eight couples of European ancestry, we found on average 1.75 genes (range 0–4) where both parents were heterozygous for rare potentially deleterious variants. A proof-of-principle study detected heterozygous *DYNC2H1* variants in a couple whose five fetuses had short-rib polydactyly. Prospective analysis of two couples with multiple pregnancy terminations for fetal akinesia syndrome was performed and a diagnosis was obtained in both the families. The first couple were each heterozygous for a previously reported *GLE1* variant, p.Arg569His or p.Val617Met; both were inherited by their two affected fetuses. The second couple were each heterozygous for a novel *RYR1* variant, c.14130-2A>G or p.Ser3074Phe; both were inherited by their three affected fetuses but not by their unaffected child. Biallelic *GLE1* and *RYR1* disease-causing variants have been described in other cases with fetal akinesia syndrome. We conclude that exome sequencing of parental samples can be an effective tool for diagnosing lethal recessive disorders in outbred couples. This permits early prenatal diagnosis in future pregnancies.

## Introduction

Rare disorders that result in antenatal or neonatal death are both phenotypically and genetically heterogeneous. Diagnosing lethal fetal disorders has previously been very difficult because of the large number of potential genes, the phenotypic variability associated with many known genetic causes and the challenges of defining phenotype and pathology in a mid-gestation fetus. For couples with multiple affected fetuses, most result from autosomal recessive mutations with a 25% recurrence risk for each future pregnancy. Current testing of a limited number of genes guided by phenotype often fails to achieve a molecular genetic diagnosis. Consequently, early prenatal testing is not possible and ultrasound diagnosis in the second trimester is the only potential option.

Exome sequencing is a powerful tool for both disease gene identification and diagnosis of monogenic disorders. Autosomal recessive diseases can be investigated by a combination of autozygosity mapping and exome sequencing in consanguineous pedigrees to identify homozygous pathogenic variants (reviewed by Gilissen *et al*^1^). In outbred families, a novel disease gene can be revealed in a single affected individual through the detection of compound heterozygous variants.^2, 3^

The quantity and/or quality of DNA extracted from a fetus following a mid-gestation termination is often limited and may be inadequate for exome sequencing. Therefore, we investigated a strategy of exome sequencing DNA from the unaffected parents and applying a set of filtering criteria to identify genes where both partners are heterozygous for a potentially pathogenic variant. We first analyzed the exomes of eight unrelated couples to determine the number of genes in which potentially deleterious heterozygous variants would be detected in both partners using filtering criteria to select only rare protein-damaging or known disease-causing variants. We then performed a proof-of-principle study by testing a couple who had terminated five pregnancies due to short-rib polydactyly. Finally, we prospectively investigated two couples in the context of a current pregnancy. Both couples had undergone multiple pregnancy terminations due to severe fetal malformations that included fetal akinesia, joint contractures, hydrops, multiple pterygia and pulmonary hypoplasia. A search of OMIM revealed 182 genes in which pathogenic variants have been reported to cause at least one of these features. Previous genetic testing of some of these genes had failed to provide a genetic diagnosis. We undertook exome sequencing in these couples and then tested for cosegregation of likely pathogenic variants in their affected fetuses.

## Materials and Methods

### Subjects and phenotypic characteristics

First, the number of genes likely to yield heterozygous non-synonymous-coding or splice-site variants was assessed in eight unrelated couples of European ancestry with no known history of a lethal autosomal recessive disorder. Second, a proof-of-principle test was performed in a (previously unpublished) family where five pregnancies were affected with short-rib polydactyly caused by compound heterozygous *DYNC2H1* variants.^4, 5^

**Figure 1:**
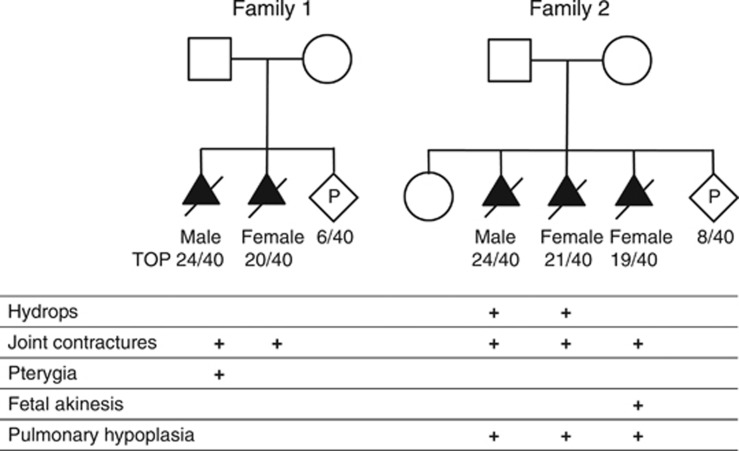
Pedigree structures and clinical characteristics of the affected fetuses.

We then prospectively investigated two unrelated couples who had terminated at least two pregnancies after an ultrasound diagnosis of a lethal disorder. X-linked inheritance was unlikely due to at least one affected fetus being female. The pedigrees and clinical characteristics of the affected fetuses are shown in Figure 1.

The first couple had two fetuses affected with fetal akinesia thought to result from a centronuclear or myotubular myopathy based on post-mortem muscle histopathology. They had no live offspring and were referred for exome sequencing 6 weeks into their third pregnancy. Karyotype and array CGH testing did not detect an abnormality, and previous testing of fetal samples had not identified deleterious variants in *CHRNG*, *BIN1* or *DNM2.* Testing of the mother for acetyl cholinesterase receptor antibodies was negative.

The second couple had a healthy daughter and then underwent three pregnancy terminations. The phenotype included joint contractures, pterygia, pulmonary hypoplasia and hydrops. Karyotype and array CGH testing did not detect an abnormality and no deleterious variants had been found in *CHRNG*, *CHRND*, *CHRNA1*, *RAPSN*, *DOK7* or *SMN1*. Exome sequencing was requested during their fourth pregnancy (8/40).

### Exome sequence analysis

Genomic DNA samples were fragmented and indexed adaptors ligated before hybridization with the Agilent SureSelect All Exon v4 capture kit (Santa Clara, CA, USA), designed to target 51 Mb of genomic sequence comprising of 20 965 genes and 334 378 exons from the CCDS, RefSeq and GENCODE databases. Paired-end 100-bp reads were sequenced on a HiSeq 2500 (Illumina, San Diego, CA, USA), 6 exomes per flow cell, to generate 80 × mean coverage with >75% of target bases at ≥30 × . Sequencing reads were separated by index, aligned with BWA-MEM to the hg19 reference genome and duplicates removed with Picard. GATK v2.7-2 was used for indel realignment, variant calling by UnifiedGenotyper and variant quality filtering on hard filters as recommended for exome data. We called variants within two pools of samples: one group included the eight healthy couples (16 samples) that we initially used to test the strategy, and the second group included the three affected families (six samples) with multiple pregnancy terminations for a presumed autosomal recessive disorder.

### Variant annotation and selection

Variants were annotated using a Linux version of Alamut-HT-1.1.11, which takes a VCF file as an input and annotates all SNVs and InDels using a range of different variant and genomic databases, including HGMD Professional. Because we were searching for variants causing a very rare lethal disorder, we applied the following criteria to look for rare potentially deleterious variants: MAF<1% in dbSNP137 variants, or MAF<0.01% in ESP or 1000 Genomes, or present in HGMD as a disease-causing variant. We excluded variants identified in 106 in-house control exomes. Variants were restricted to non-synonymous, splice site or within−50/+10 bp of flanking exons and predicted by Alamut-HT to affect splicing. Alamut-HT uses three different tools (MaxEntScan, NNSPLICE, Human Splicing Finder) in an algorithm that makes an overall prediction of potential splicing effects, namely creation of a new acceptor or donor site, activation of existing cryptic sites or an effect on the nearest splice site. We identified genes where parents either shared the same heterozygous variant (for which offspring could be homozygous) or had different heterozygous variants in the same gene (potentially compound heterozygous in offspring).

### Cosegregation studies

Putative deleterious heterozygous variants in *GLE1* or *RYR1* were confirmed in the parents by PCR/Sanger sequencing and tested in their offspring to investigate cosegregation with disease. PCR primers are available on request.

### Variant submission to locus-specific database

All confirmed variants were submitted to locus-specific databases within the LOVD 3.0 shared installation: www.LOVD.nl/DYNC2H1 (individual ID 00016506); www.LOVD.nl/RYR1 (individual ID 00011457); www.LOVD.nl/GLE1 (individual ID 00016592).

## Results

We tested a strategy to diagnose lethal autosomal recessive disorders by exome sequencing in non-consanguineous parents. Analysis of eight unrelated couples of European ancestry showed that their exomes contained an average of 103 rare heterozygous non-synonymous coding or splice-site variants (range 82–140, data not shown). The number of genes in which rare potentially deleterious heterozygous variants were identified in both members of the couple ranged from 0 to 4 (see Table 1). The average number of genes, where the couple each had a different heterozygous variant, was 1.0 and for the same rare heterozygous variant the mean was 0.75 (Table 1).

In a proof-of-principle study, we sequenced the exomes of a couple who had five fetuses affected with short-rib polydactyly caused by compound heterozygous *DYNC2H1* variants. Rare, potentially deleterious heterozygous variants were identified in both partners for two genes, *NR1D1* and *DYNC2H1* (Table 2). Exome sequencing correctly identified the *DYNC2H1* variants as c.2819-14A>G (NG_016423.1) and c.7577T>G, p. Ile2526Ser (NM_001080463.1). *In silico* tools (Alamut) suggest that the p. Ile2526Ser missense variant is deleterious, and the c.2819-14A>G variant is predicted to create a new splice acceptor site: 13 bp upstream from exon 20 (numbering according to NG_016423.1).

We then investigated two families referred to our local clinical genetics service because of a history of multiple fetuses terminated after an antenatal ultrasound scan diagnosis of fetal akinesia syndrome.

The first couple had two pregnancies affected with pterygia and/or joint contractures. Rare heterozygous missense variants in two genes, *CLIP1* and *GLE1*, were found in both partners (Table 2). Sanger sequencing confirmed that the parents were each heterozygous for a *GLE1* variant (NM_001499.2) and that both variants were present in the two affected fetuses. These *GLE1* variants, p.Arg569His and p.Val617Met, have been reported in Finnish patients with clinical phenotypes consistent with fetal akinesia syndrome.^6^

The second couple had a healthy daughter followed by three fetuses with arthrogryposis. We identified four genes in which both parents were heterozygous for rare variants with possible pathogenic consequence (Table 2). Sanger sequencing confirmed that the couple were heterozygous carriers of the novel c.14130-2A>G (NG_008866.1) and c.9221C>T (p.Ser3074Phe) *RYR1* variants (NM_000540.2). All three affected fetuses were found to be compound heterozygotes and the couple's unaffected daughter had not inherited either variant. The c.14130-2A>G variant affects a conserved splice acceptor site, but p.Ser3074Phe is a novel missense variant. Assuming a very rare autosomal recessive disorder, where the probability of affected and unaffected offspring is 0.25 and 0.75, respectively, we calculated the maximal LOD score in the four offspring (three affected and one unaffected) as −log_10_(0.25^3^ × 0.75)=1.9.

## Discussion

We developed a strategy to diagnose rare autosomal recessive lethal disorders by exome sequencing in non-consanguineous couples with a history of multiple affected fetuses. The aim was to obtain a molecular genetic diagnosis and enable prenatal testing in current/future pregnancies. A proof-of-principle study successfully identified heterozygous *DYNC2H1* variants causing short-rib polydactyly. Two families presenting with a current at-risk pregnancy were then studied prospectively, and a molecular genetic diagnosis was obtained in both families through the identification of *GLE1* and *RYR1* variants causing a severe form of fetal akinesia syndrome with arthrogryposis.

The two *GLE1* missense variants have already been reported in separate Finnish families who also carry the Fin(Major) c.432-10A>G variant that creates an illegitimate splice acceptor site; one patient compound heterozygous for p.Arg569His and Fin(Major) was affected with lethal congenital contracture syndrome type 1 (OMIM 253310), while six patients compound heterozygous for p.Val617Met and Fin(Major) had lethal arthrogryposis with anterior horn disease (OMIM 611890).^6^ Interestingly, the frequency of Fin(Major) is up to 2% in Finnish sub-populations,^6^ while p.Arg569His (rs121434407) has frequency of 0.03% in the ESP database commonly used for variant frequency filtering, owing to the large number of Finnish samples contributing to this project. We conclude that compound heterozygosity for these known *GLE1* variants is the cause of fetal akinesia syndrome in this family.

Heterozygous variants in the *RYR1* gene are a known cause of susceptibility to malignant hyperthermia.^7^ There is no history of malignant hyperthermia in either the parents or wider members of family 2 but penetrance of this condition is known to be low. Dominant or recessive *RYR1* mutations are a common cause of congenital myopathies.^8, 9^ There is considerable phenotypic variability reported and a small subset have a more severe form characterized by a fetal akinesia syndrome,^10, 11^ including one fetus with compound heterozygous *RYR1* missense variants that was terminated at 32 weeks' gestation.^10^ Cosegregation of the c.14130-2A>G and p.Ser3074Phe *RYR1* variants in family 2 is consistent with linkage (maximal LOD score 1.9, the threshold for suggestive linkage). We conclude that these *RYR1* variants are highly likely to be the cause of fetal akinesia, arthrogryposis and pulmonary hypoplasia in all three fetuses.

Ultrasound analysis at 20 weeks for the on-going pregnancies showed no evidence of fetal akinesia syndrome. Couple 2's *RYR1* variants were confirmed shortly before this scan and they were offered the option of prenatal testing by amniocentesis sampling. In view of the normal ultrasound scan, they decided not to risk a miscarriage by undergoing an invasive procedure. Both couples have indicated that they would seek early prenatal testing in future pregnancies.

Our strategy of exome sequence analysis in the parents of affected fetuses generated a very small number of potential pathogenic variants for confirmation and cosegregation analysis by Sanger sequencing. Several studies have diagnosed recessive disease through the identification of compound heterozygous variants by exome sequencing in a single individual.^12, 13^ We considered the alternative approach of sequencing the exome of one of the fetal DNA samples, but there is a higher risk of exome sequencing failure as the quality of DNA extracted from terminated fetuses can be poor compared with leukocyte DNA from healthy adults, particularly if the only source of fetal DNA is formalin-fixed paraffin-embedded tissue. In addition, the amount of fetal DNA available may be insufficient for exome sequencing or limited in quantity such that it is prudent to sequence the parents and save the precious fetal sample for confirming potential pathogenic variants or for future studies. For the couple with *RYR1* variants, cell culture was required to obtain DNA for cosegregation studies in two of the fetuses. This took 3 weeks, by which time the potentially pathogenic variants had been identified through analysis of the parental exome sequence data.

We conclude that exome sequencing in non-consanguineous couples can provide an effective means of making a genetic diagnosis of lethal autosomal recessive disorders. Diagnosing lethal fetal disorders has previously been very difficult because of the large number of potential genes, the phenotypic variability associated with many known genetic causes and the challenges of defining phenotype and pathology in a mid-gestation fetus. Sequencing parental samples overcomes issues of limited quality or quantity of fetal samples. A genetic diagnosis confirms the risk for future offspring and permits early prenatal diagnosis or preimplantation genetic diagnosis in future pregnancies. This in turn reduces the anxiety associated with waiting until mid pregnancy for an ultrasound diagnosis, and avoids the added distress of a late termination of pregnancy. The strategy is also applicable to those disorders not detectable by ultrasound diagnosis where late fetal demise or a neonatal death could not otherwise be predicted.

## Figures and Tables

**Table 1:** Genes with rare heterozygous non-synonymous or splicing variants identified by exome sequencing in both partners of eight non-consanguineous couples of European ancestry

*Couple*	*Number of genes with a different heterozygous variant*	*Genes*	*Number of genes with the same heterozygous variant*	*Genes*
1	2	SPTBN4, ALDOB	2	LY6G5C, SYNPO2
2	3	C5orf42, SUPT20H, ZFHX3	0	
3	0		1	LENG8
4	0		1	CUL7
5	0		0	
6	0		2	KCNMA1, PRR12
7	0		0	
8	3	HSPG2, MUC16, ZNF469	0	
Mean	1.0		0.75	

**Table 2:** Genes with rare heterozygous non-synonymous or splicing variants identified by exome sequencing in three couples with multiple fetuses affected with a presumed autosomal recessive lethal condition

*Family*	*Number of genes with a different heterozygous variant in each parent*	*Genes*	*Number of genes with the same heterozygous variant in each parent*
Short-rib polydactyl	2	**DYNC2H1**, NR1D1	0
1	2	CLIP1, **GLE1**	0
2	4	CC2D1, FAM129C, OBSCN, **RYR1**	0

Genes shown in bold are those in which compound heterozygous variants were identified in the affected fetuses.
